# Supplementary material for: Nanoscatterer-Assisted Fluorescence Amplification Technique
Source: Nanomaterials (Basel). 2023 Oct 30;13(21):2875. doi: 10.3390/nano13212875 (PMC10648225; doi:10.3390/nano13212875)
Supplement: Supplementary file 1 [file nanomaterials-13-02875-s001.zip › nanomaterials-2647538-supplementary.pdf]

Supplementary Materials: Nanoscatterer-Assisted Fluorescence Amplification Technique

Sylvain Bonnefond [1,†], Antoine Reynaud [2], Julie Cazareth [2], Sophie Abélanet [2], Massimo Vassalli [3], Frédéric Brau [2,‡] and Gian Luca Lippi [1,*,‡]

1. Dynamic Light Scattering

We recall the background of Dynamic Light Scattering for the estimate of the hydrodynamic radius R_h.

D_s is measured indirectly from the particle intensity correlation curve, whose exponential decay [1] can be approximated by the first two cumulants of the measured distribution [2] since the large statistics ensure convergence to a Gaussian. Under these conditions, the Siegert relation holds for $g^{(2)}$ [3]:

$$g^{(2)}(\tau) = B + (|G(\tau)|)^2\,, \tag{S1}$$

where B is the so-called baseline, τ is the delay time and $G(\tau)$ is the field correlation function. This last function, for homogeneous spherical particles, is:

$$G(\tau) = \int_0^\infty c(\Gamma)e^{-\Gamma\tau}d\Gamma \quad , \quad q = \frac{4\pi n_{H_2O}}{\lambda}\sin\frac{\theta}{2}\,, \tag{S2}$$

where $\Gamma = q^2 D_s$ is the decay rate, $c(\Gamma)$ is the weighted distribution of decay rates for the normalized intensity, q is the modulus of a scattering vector determined by the solvent refractive index n, the laser wavelength ($\lambda = 680nm$), and θ is the angle of detection ($\theta = 90°$ relative to the direction of the incident beam). As the NPs undergo Brownian motion, R_h is obtained directly from D_s using the Stokes–Einstein equation[1]:

$$R_h = \frac{kT}{6\pi D_s \mu}\,, \tag{S3}$$

where k is the Boltzmann constant, T the absolute temperature, and μ is the dynamic solvent viscosity ($\mu_{H_2O} = 1mPa \cdot s$ at room temperature) (Figure 1d of main paper) [4]).

2. Pump laser

As a pump source for the fluorophore, we use a Q-switched Nd:YAG laser (Quanta-Ray INDI-40-10-HG, Spectra-Physics), which generates pulses with energy $\sim$ 120 mJ at $\lambda_{IR} = 1.064\mu$m, at a repetition rate $\nu_p = 10$ Hz. The IR pulses are converted by an intracavity harmonic generator, followed by a separator, to produce pulses with duration $\tau_p \approx .5$ ns and energy $\sim$ 30 mJ at $\lambda_{UV} = 355nm$. The latter pump a tunable Optical Parametric Oscillator (OPO) for parametric downconversion (OPO, VersaScan/120/MB – manufactured by GWU) based on a beta-barium Borate (BBO) crystal. The OPO is tuned to emit a signal at $\lambda_P = 490$ nm, which is the optimum absorption wavelength of the of the FITC dye.

3. Filters calibration

The ND filters F1 must be calibrated to establish the actual relationship between the actual pump energy sent to the sample and that recorded by the by the photodiode P1. This is performed in two steps: (1) calibration of the actual OD of each filter F1 with our pump pulses at $\lambda_p = 490$ nm; (2) calibration of the actual energy reaching the sample (described in the main paper) against the measured energy at P1 in the absence of filters F1 (i.e., calibration of the entire experimental line). The combination of these two steps gives the actual pump energy delivered to the sample for each pulse measured by P1.

Table S1. Calibration of the Kodak Wratten II OD filters F1 with experimentally determined OD with our pump pulses at $\lambda_p = 490$ nm.

Nominal OD	Experimental OD
0.30	0.430 ± 0.004
0.50	0.638 ± 0.000
0.70	0.810 ± 0.001
0.80	0.942 ± 0.001
0.90	1.076 ± 0.001
$1.10 = 0.30 + 0.80$	1.369 ± 0.001
$1.50 = 0.70 + 0.80$	1.739 ± 0.002

An energy meter (PE25BF-C, Ophir) is used to measure the pulse energy for both steps. Each calibration is averaged over 100 pulses. For filters F1 (step 1), we obtain the experimental ODs given in the right column of Table S1 (with standard deviation) corresponding to the nominal OD given by the manufacturer.

4. Temperature increase in sample

An estimate of the temperature increase in the sample can be made by assuming that the energy of the quantum defect [5] is completely absorbed and converted into internal energy.

The quantum defect is

$$E_{qd} = \hbar(\omega_L - \omega_F)\,, \tag{S4}$$

where ω_L and ω_F stand for the laser and fluorescence frequencies, respectively. Quantitatively, the energy fraction ϵ_{qd} is

$$\epsilon_{qd} = \frac{E_{qd}}{E_L} \tag{S5}$$

$$= \frac{\lambda_F - \lambda_L}{\lambda_L} \tag{S6}$$

$$\approx \frac{30nm}{490nm} \tag{S7}$$

$$\approx 0.06\,, \tag{S8}$$

and thus, we estimate that approximately 6% of the energy incident on the sample is converted into heat. The amount of energy absorbed by the sample is therefore $E_a \leq 1.8 \times 10^{-4} J$ per pulse.

We will focus on the highest energy pulses with the highest NP concentrations at 200 μm FITC dilution, where the pump beam is almost completely absorbed by the cell volume (thickness 2 mm). For all other conditions, the absorbed fraction is lower and therefore the corresponding temperature increase is also lower.

Due to the geometric shape of the laser pulses coming from the OPO, the volume in which the energy is deposited is (see Section 3.1 of the main paper)

$$V_a = w \times h \times d\,, \tag{S9}$$

$$\approx 200\mu m \times 500\mu m \times 2000\mu m\,, \tag{S10}$$

$$\approx 2 \times 10^{-10} m^3\,. \tag{S11}$$

The relationship between the temperature rise ΔT and absorbed energy is

$$\Delta T = \frac{E_a}{C \cdot M}\,, \tag{S12}$$

where C is the specific heat capacity and M is the mass. Since the dominant mass is that of water, we find that in the irradiated volume, we have

$$M = 10^3 Kgm^{-3} \times 2 \times 10^{-10} m^3 , \quad \text{(S13)}$$
$$= 2 \times 10^{-7} Kg \quad \text{(S14)}$$

with $C = 4187 J \cdot Kg^{-1} \cdot K^{-1}$. Substituting into Eq. (S12), we obtain:

$$\Delta T \approx \frac{1.8 \times 10^{-4} J}{2 \times 10^{-7} Kg \cdot 4187 J\, Kg^{-1}\, K^{-1}} , \quad \text{(S15)}$$
$$\approx 0.2K \quad \text{(S16)}$$

which represents the increase in temperature per pulse (worst case). For the 10 pulse sequence used in the experiment, we can expect a maximum $2K$ temperature increase if we completely neglect the convection processes spontaneously induced by the heat gradient. The temperature increase thus remains quite moderate, in agreement with the findings [6] of very good cell survival in a laser configuration. This is a meaningful test because cells are very sensitive to temperature changes. We can therefore expect that in most experiments, the effect of heating by the quantum defect will be negligible.

1. Berne B.J. and Pecora R. *Dynamic light scattering: with applications to chemistry, biology, and physics*. Dover Publications Inc., Mineola, USA, 2000.
2. Koppel D.E. Analysis of macromolecular polydispersity in intensity correlation spectroscopy: the method of cumulants. *The J. Chem. Phys.* **1972**, *57* 4814–4820.
3. Brown W. *Dynamic light scattering: the method and some applications*, vol. 313. Clarendon, Oxford, UK, 1993.
4. Korson L., Drost-Hansen W., and Millero F.J. Viscosity of water at various temperatures. *The J. Phys. Chem.* **1969**, *73* 34–39.
5. Paschotta, R. Quantum defect. *RP Photonics Encyclopedia*. Available online: https://www.rp-photonics.com/quantum_defect.html (accessed 22 October 2023).
6. Gather M.C., and Yun S.H. Single-cell biological lasers. *Nat. Photonics* **2011**, *5* 406–410.
